# Gut microbiota-derived imidazole propionate is associated with obesity

**DOI:** 10.3389/fnut.2026.1861257

**Published:** 2026-08-03

**Authors:** Linwei Li, Chen Wang, Luyao Liu, Ting Xu, Xiaoqun Nie, Yujie Liu, Hongwei Zhang, Chen Yang, Jianzhong Di

**Affiliations:** 1 Shanghai Sixth People’s Hospital Affiliated to Shanghai Jiao Tong University School of Medicine, Shanghai, China; 2 State Key Laboratory of Microbial Metabolism, School of Life Sciences & Biotechnology, Shanghai Jiao Tong University, Shanghai, China

**Keywords:** bariatric surgery, gut microbiota, imidazole propionate, metabolite, obesity

## Abstract

Obesity is a progressive metabolic disorder with some well-recognized markers, such as increased or elevated branched-chain amino acids (BCAAs). However, the role of gut microbiota-derived metabolites remains unknown in Asian populations. By employing an integrated multi-omics approach combining metagenomic and plasma metabolomic profiling in an Asian cohort alongside a longitudinal analysis of a bariatric surgery subgroup. We identified a distinct metabolic signature in obesity characterized by depleted circulating histidine and a concomitant elevation of Imidazole Propionate (ImP). The elevated ImP level not only positively correlated with the body mass index (BMI) but also increased progressively across obesity severity categories, and were associated with the taxonomic enrichment of ImP-producing species, such as *Streptococcus mutans* and *Lactobacillus gasseri*. Meanwhile, the ImP level showed rapid reduction within 3 months post-bariatric surgery. Collectively, our findings indicate that gut dysbiosis and histidine metabolism toward ImP production link with obesity and metabolic dysfunction.

## 1 Introduction

Obesity, together with its associated metabolic disorders, constitutes one of the leading causes of death worldwide, with prevalence rising unabated for three decades. The 2025 World Obesity Atlas projects that by 2030, 50% of the world’s adults will be overweight or obese (1). Obesity is not only a metabolic and social issue, but also closely associated with other diseases, including Type 2 Diabetes (T2D), cardiovascular diseases, and even cancer (2). Etiologically, obesity is multifactorial, integrating genetic susceptibility, sociodemographic context, psychological aspects, and dietary pattern dimensions (3). Within this complex etiological framework, the gut microbiome has emerged as a critical environmental factor that modulates host metabolic phenotypes (4).

The gut microbiota plays an important role in digestion and absorption, energy homeostasis, and lipid and bile acid metabolism (5). Consequently, the dysregulation of the gut microbiota is associated with many diseases, such as obesity, diabetes, and cardiovascular diseases (6). Critically, metagenomic studies have established that distinct microbial assemblies differentiate patients with obesity from lean individuals, characterized by significant shifts in alpha and beta diversity (7, 8). Beyond its structural composition, the gut microbiome possesses a vast genetic repertoire that exceeds the human genome by nearly 1,000 fold, functioning as a sophisticated biochemical bioreactor (9). This expansive enzymatic capacity allows the microbiota to transform dietary and endogenous substrates into a myriad of small-molecule metabolites that serve as the functional read-out of host–microbe interactions (10, 11). In individuals with obesity, the reorganization of this collective metabolic potential leads to a reshaped systemic metabolite profile. This includes not only the production of short-chain fatty acids (SCFAs) but also a diverse array of bioactive compounds such as ammonia, phenols, and indole derivatives, which can translocate into the host circulation to modulate physiological processes (12, 13).

However, beyond the well-characterized metabolites (7, 14), the role of many other microbial catabolites, particularly those derived from under-explored pathways, in obesity and its metabolic sequelae remains largely unknown. Furthermore, whether these microbial-metabolic axes exhibit dynamic reversibility following clinical interventions, such as bariatric surgery, necessitates more comprehensive, longitudinal investigation. To address these gaps, we utilized both untargeted and targeted plasma metabolomic profiling alongside shotgun metagenomic sequencing to decipher the functional output of the obese gut environment. By incorporating a longitudinal follow-up of a sub-cohort of 24 patients who underwent laparoscopic sleeve gastrectomy (LSG), we evaluated the stability of metabolic shifts.

## 2 Materials and methods

### 2.1 Study participants

The clinical cohort included 104 patients with obesity and 76 healthy controls recruited at Shanghai Sixth People’s Hospital. Patients with obesity were recruited from the Department of Bariatric and Metabolic Surgery and were eligible if they were adults aged 18–65 years with a BMI ≥ 28 kg/m^2^ according to the Chinese diagnostic criteria for obesity. Healthy controls were recruited during the same study period according to predefined criteria and were eligible if they were adults aged 18–65 years with a normal BMI range of 18.5–24.0 kg/m^2^, regular lifestyle and dietary habits, and no known major chronic diseases or obesity-related metabolic disorders.

For both groups, individuals were excluded if they had acute illness; had used antibiotics, probiotics, health products, or nutritional supplements within four weeks before sample collection; had gastrointestinal disease or a history of gastrointestinal surgery; were pregnant or lactating; had undergone major surgery within the previous three months; or had other conditions considered unsuitable for participation by the investigators. All participants provided written informed consent before enrolment.

Among the 180 enrolled participants, 163 participants, including 90 patients with obesity and 72 healthy controls, provided plasma samples of sufficient quality for metabolomic analysis. A total of 154 participants, including 78 patients with obesity and 76 healthy controls, provided fecal samples for metagenomic sequencing. The remaining participants were excluded from the corresponding omics analyses because of insufficient sample volume or failure to meet quality-control criteria. Overall, 136 participants completed both metabolomic and metagenomic profiling. In addition, a longitudinal sub-cohort of 24 patients with obesity underwent laparoscopic sleeve gastrectomy and provided paired plasma samples at baseline and 3 months postoperatively.

### 2.2 Clinical data and sample collection

Baseline information, including age, gender, height, weight, and BMI, was collected from both two groups. Participants fasted overnight for at least 12 h, and whole blood samples were collected in the morning. Plasma samples after centrifugation were stored at −80 °C until analysis. Clinical parameters, including total cholesterol (TC), triglycerides (TG), high-density lipoprotein cholesterol (HDL-C), low-density lipoprotein cholesterol (LDL-C), fasting plasma glucose (FPG), fasting insulin (FINS), glycated hemoglobin (HbA1c), alanine aminotransferase (ALT), aspartate aminotransferase (AST) and *γ*-glutamyltransferase (GGT) were measured using standard clinical assays. For the surgical group, clinical data and plasma samples were collected at baseline and 3 months postoperatively. Without any antibiotic treatment or probiotic-containing foods for 1 month prior to sample collection, fecal samples were collected and stored frozen at −80 °C until analysis.

### 2.3 Plasma metabolomics analysis

#### 2.3.1 Sample preparation and LC–MS conditions

Plasma samples were thawed on ice. A volume of 50 μL of each sample was mixed with 150 μL of methanol. After vortexing for 1 min, the mixtures were centrifuged at 14,000 × *g* for 15 min, and the resulting supernatant was collected for Liquid Chromatograph Mass Spectrometer (LC–MS) analysis. Quality control samples were prepared by combining aliquots of plasma from a subset of the cohort and processed following the identical procedure as the experimental samples.

Untargeted metabolomic profiling was conducted using an ultra-high-performance liquid chromatography system (UltiMate 3,000, Thermo Fisher) coupled to a Q Exactive hybrid quadrupole-Orbitrap mass spectrometer (Thermo Fisher). All study samples were injected in a randomized order. Throughout the analytical run, QC samples and blank samples (80% methanol in water) were injected at regular intervals. The injection volume was 1 μL.

Metabolite separation was achieved using an XSelect HSS T3 column (100 mm × 3 mm, 2.5 μm, Waters) maintained at 45 °C, with a mobile phase flow rate of 0.4 mL/min. The mobile phases consisted of solvent A (0.1% formic acid in water) and solvent B (0.1% formic acid in methanol). The gradient elution program was as follows: 0% B, 0 min; 50% B, 10 min; 98% B, 14 min; held at 98% B until 16 min.

Mass spectrometric detection was performed in electrospray ionization positive mode. Key instrument parameters were set as follows: sheath gas pressure, 40 arbitrary units; capillary temperature, 320 °C. Full-scan MS data were acquired over a mass range of m/z 70–1,050 with a resolution of 70,000.

#### 2.3.2 Untargeted data processing by LC–MS

Raw LC–MS data were processed using Progenesis QI software (version 3.0, Waters). Initial metabolite annotation was performed with MetaboScape (Version 1.0.7951). Subsequently, features with associated MS/MS spectra were subjected to fragment ion matching using a local version of the Biomolecules Library, which was integrated as a plugin within Progenesis QI. The search parameters were configured as follows: for the initial MetaScope annotation, a precursor mass tolerance of 12 ppm, a retention time tolerance of 0.1 min, and a fragment mass tolerance of 12 ppm were applied; for the subsequent Biomolecules Library search, more stringent tolerances of 5 ppm for both precursor and fragment masses were used.

#### 2.3.3 Targeted analysis by LC–MS

Targeted quantification of amino acid metabolites was performed using the same LC–MS platform (UltiMate 3,000 coupled to Q Exactive). Chromatographic separation was achieved on an XSelect HSS T3 column (100 mm × 3 mm, 2.5 μm, Waters) maintained at 45 °C. The mobile phase consisted of 0.1% formic acid in water (aqueous phase) and 0.1% formic acid in methanol (organic phase), with a flow rate of 0.4 mL/min under gradient elution conditions. Data acquisition and processing, including peak integration and quantification, were performed using Xcalibur software (version 4.4.0, Thermo Fisher).

### 2.4 Fecal metagenomic analysis

Fecal DNA was extracted from 154 samples. Shotgun metagenomic sequencing was performed on the Illumina HiSeq for paired-end sequencing. Raw data (FASTQ format) underwent quality control using cutadapt (v1.9.1) to remove adapters and low-quality sequences (base quality < 20, N content > 10%, or length < 75 bp). Taxonomic profiling was performed using Kraken2 (v2.0.9; RRID: SCR_005484) with the standard database comprising the NCBI RefSeq genomic archive (>30,000 genomes). The relative abundance of microbial species was then estimated and refined using Bracken2 (Bayesian Reestimation of Abundance with KrakEN; RRID: SCR_018902), which employs a Bayesian statistical model to correct for classification biases.

UrdA-related genes were identified from the functional annotation results generated from the shotgun metagenomic sequencing data. Genes annotated as UrdA or UrdA homologs in the functional gene annotation table were extracted and used for downstream analysis. The normalized abundance of UrdA-related genes was compared between individuals with obesity and healthy controls. In addition, taxa previously reported to carry Y- or M-type UrdA homologs were summarized, and their associations with circulating ImP levels were assessed using Spearman correlation analysis.

### 2.5 Statistical analysis

All statistical analyses were conducted in R (version 4.5.2). The statTarget package was used for quality control of the targeted metabolomics data. OPLS-DA and PCoA were used to assess overall phenotypic differences between the two groups. Differential metabolite screening used the limma package to construct a linear regression model, correcting for confounding factors such as age and sex; differential microbial community analysis used the ANCOM-BC package for abundance difference tests. Wilcoxon rank-sum tests were used for comparisons between the two groups. Correlation analysis used Spearman correlation coefficients, with an FDR < 0.05 as the threshold for multiple testing to screen for significant associations. Finally, based on the correlation results, Cytoscape (version 3.1.4; RRID: SCR_003032) software was used to construct a multi-omics association network.

## 3 Results

### 3.1 Clinical characteristics of the study participants

A total of 180 participants were enrolled, involving 104 patients with obesity and 76 healthy people. While gender distribution was comparable between groups, the obesity group was slightly older (*p* = 0.035). Patients with obesity exhibited a significantly impaired metabolic profile compared to controls, characterized by elevated BMI, TC, LDL-C, FPG, and FINS, along with lower HDL-C levels (Table 1).

**Table 1:** Summary of clinical parameters of the study participants.

Variable	Group		*p*-value^2^
	Obesity *N* = 104^1^	Healthy *N* = 76^1^	
Age	32.79 ± 9.33	29.58 ± 10.48	0.035
Sex			0.341
Female	70 (67.3%)	57 (75.0%)	
Male	34 (32.7%)	19 (25.0%)	
Weight (kg)	114.83 ± 23.09	58.63 ± 7.92	<0.001
BMI (kg/m^2^)	40.01 ± 6.41	21.33 ± 1.71	<0.001
TC (mmol/L)	5.08 ± 0.99	4.66 ± 1.00	0.006
TG (mmol/L)	1.87 ± 1.33	1.63 ± 0.70	0.162
HDL-C (mmol/L)	1.15 ± 0.40	1.94 ± 0.43	<0.001
LDL-C (mmol/L)	3.34 ± 1.05	2.96 ± 0.96	0.015
FPG (mmol/L)	6.08 ± 2.03	5.45 ± 0.67	0.010
FINS (μU/ml)	37.59 ± 23.19	11.84 ± 3.34	<0.001
HbA1C (%)	6.26 ± 1.26		
HOMA-IR	10.35 ± 7.87	2.86 ± 0.80	<0.001

1 Mean ± SD or *n* (%), as appropriate.

2 *p*-value were derived from Student’s *t*-test for continuous variables and Person’s chi-square test for categorical variables. A two-tailed *p*-value < 0.05 was considered statistically significant.

### 3.2 Obesity is associated with distinct plasma metabolomic signatures with imidazole propionate elevated

**Figure 1:**
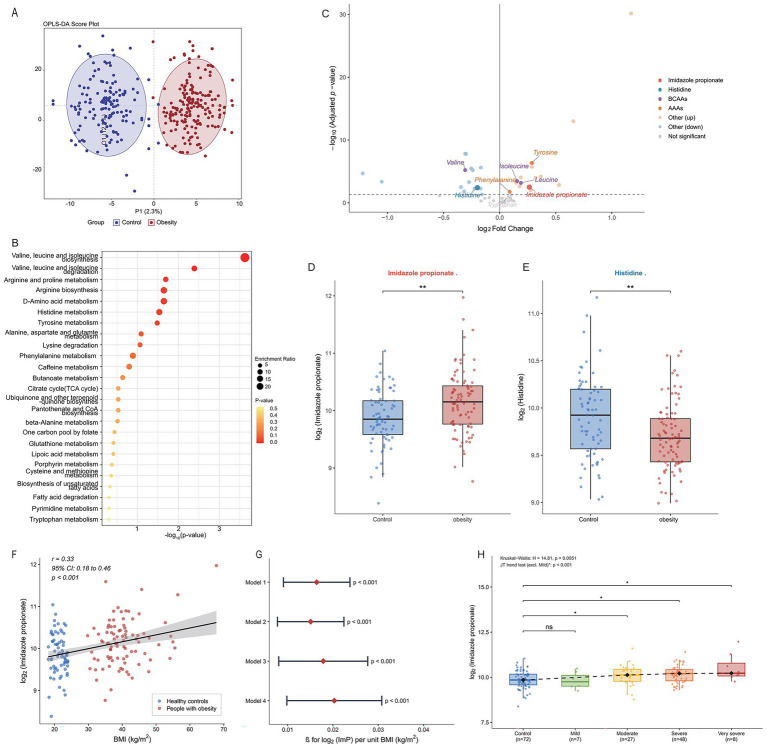
Metabolic profiling differences and ImP as a key microbiota-derived metabolite associated with obesity. **(A)** OPLS-DA score plot of untargeted metabolite between two groups. **(B)** Pathway enrichment analysis was conducted via the MetaboAnalyst 6.0 platform (https://www.metaboanalyst.ca/). Differentially expressed metabolites were mapped against the KEGG (RRID: SCR_012773) to identify significantly enriched metabolic pathways. **(C)** Volcano plot of differential metabolites. The volcano plot displays significantly altered metabolites between groups using the limma linear model, with adjustments for sex and age (Benjamini–Hochberg adjusted *p* < 0.05). **(D,E)** Elevation of circulating ImP and depletion of circulating histidine in obesity. Center lines represent the median; whiskers represent the range. **p < 0.01 by Wilcoxon rank-sum test. **(F)** Scatter plot of the correlation between ImP and BMI. Pearson correlation analysis used. **(G)** Robustness of the ImP-BMI association. Forest plot showing the regression coefficients (*β*) and 95% CIs for log_2_(ImP) per unit increase in BMI (kg/m^2^) across four adjustment models. **(H)** Differences in plasma ImP levels across obesity grades. It were assessed using the Kruskal–Wallis rank-sum test, followed by Dunn’s test with Benjamini–Hochberg correction for multiple comparisons against the healthy control group. To evaluate the presence of a monotonic dose–response relationship, the Jonckheere–Terpstra trend test was applied to four ordered groups.

To characterize the global metabolic alternations associated with obesity, we performed untargeted plasma metabolomic profiling. OPLS-DA revealed a clear separation between groups (*p* < 0.05), confirming distinct metabolic signatures (Figure 1A). Pathway enrichment analysis on the significantly different metabolites indicated that multiple altered amino acid metabolic pathways in people with obesity, including valine, leucine and isoleucine biosynthesis and degradation, arginine, histidine and tyrosine metabolism (Figure 1B).

Targeted analysis confirmed broad amino acid dysregulation (Figure 1C). Several amino acids and derivatives were significantly elevated in people with obesity, including glutamate, phenylalanine, tyrosine, leucine, isoleucine, kynurenic acid, and serotonin, consistent with prior reports of branched-chain and aromatic amino acid accumulation in metabolic disease (15). In contrast, histidine, arginine, glutamine, serine, and asparagine were significantly depleted. Correlation analysis revealed the broader clinical relevance of these metabolic shifts (Supplementary Figure S1A). Metabolites elevated in people with obesity showed positive correlations with TC and FPG, while depleted metabolites showed the opposite pattern.

Notably, in parallel with reduced histidine, we detected a significant elevation of ImP, which derives from histidine metabolism by gut microbiota, in people with obesity (*p* < 0.01; Figures 1D,E). Correlation analysis across the full metabolomic cohort revealed a significant positive association between plasma Imp and BMI (*r* = 0.33, *p* < 0.001; Figure 1F). To evaluate the robustness of this association, we performed sequential multivariable regression adjusting for potential confounders. The positive association remained statistically significantly after adjustment for age and sex (Model 2), further adjustment for blood lipids including TC, TG and HDL (Model 3), and additional adjustment for HOMA-IR (Model 4; Figure 1G).

To further delineate the relationship between obesity and ImP, we stratified the full metabolomic cohort according to the Chinese clinical criteria: normal weight (BMI 18.5–24.0 kg/m^2^), mild obesity (BMI 28.0–32.5 kg/m^2^), moderate obesity (BMI 32.5–37.5 kg/m^2^), severe obesity (BMI 37.5–50.0 kg/m^2^), and very severe obesity (BMI > 50.0 kg/m^2^). Plasma ImP levels increased progressively across obesity severity categories, with a statistically significant monotonic trend (Jonckheere–Terpstra trend test, *p* < 0.001; Figure 1H). Post-hoc Dunn’s tests with BH correction confirmed significant elevations in the moderate, severe and very severe obesity groups relative to healthy controls, while the mild obesity group (*n* = 7) showed no difference. Taken together, these data establish that plasma ImP elevation not only relates to the obesity but also is a graded feature of the obese state.

### 3.3 The gut microbiome–derived imidazole propionate links microbial composition to obesity

**Figure 2:**
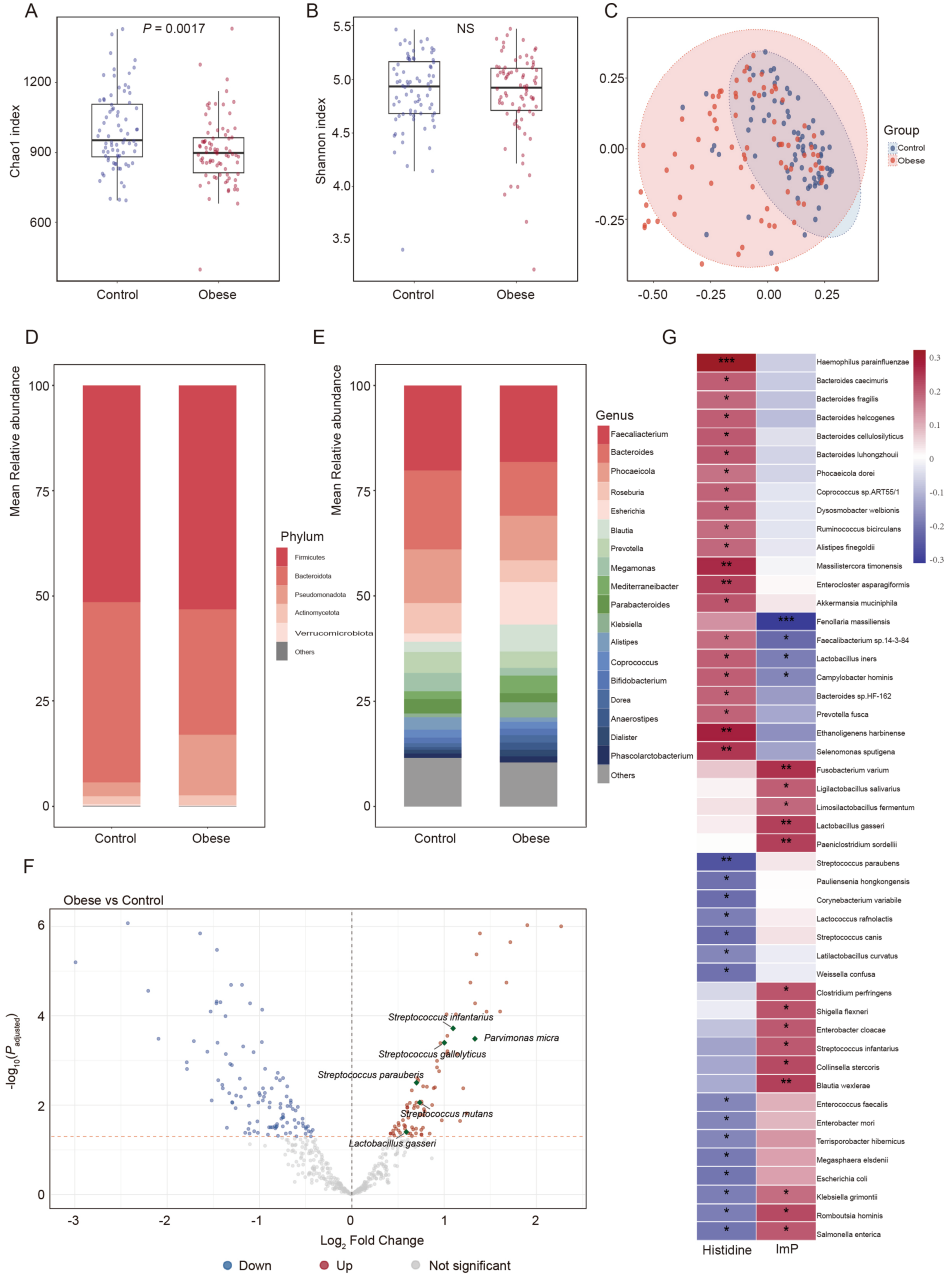
Gut microbiota alterations and ImP-related bacterial enrichment in obesity. **(A)** Chao 1 index of obesity patients and controls. **(B)** Shannon diversity index of obesity patients and controls. **(C)** Beta diversity of the genera analyzed by Bray–Curtis dissimilarity PCoA. **(D)** Taxonomic composition at the phylum levels. **(E)** Taxonomic composition at the genus levels. **(F)** Volcanic map of differential species. The horizontal coordinate represents the fold change (log2FoldChange) of species in different groups, and the vertical coordinate represents the significance level of the difference (-log10P adjusted). Each point in the volcano map represents a species, in which the species with significantly up-regulated expression are represented by red dots, and those with significantly down-regulated expression are represented by blue dots. **(G)** Cross-correlation analysis between microbiota and histidine and ImP. The vertical coordinate represents the differentially abundant bacteria at the species level, and the horizontal coordinate represents the histidine and ImP levels. Colors indicate the direction and strength of Spearman correlations. Red indicates a positive correlation, while blue indicates a negative correlation. Asterisks denote statistical significance (*p < 0.05; **p < 0.01; ***p < 0.001).

Given that ImP originates exclusively from gut microbiota, we performed metagenomic sequencing. Diversity analysis revealed significant dysbiosis in obesity. The chao1 index was markedly lower in the obese group (*p* = 0.0017) (Figure 2A), indicating reduced species richness, whereas the Shannon index showed no significant difference (Figure 2B), in agreement with previous researches (16, 17). PCoA based on Bray–Curtis dissimilarity demonstrated a distinct clustering of microbial communities by group (*R*^2^ = 0.051, *p* < 0.001) (Figure 2C). PERMDISP analysis further indicated a significant difference in within-group dispersion between the two groups (*F* = 8.32, *p* = 0.004), with individuals with obesity showing greater dispersion than healthy controls. These findings suggest that obesity-associated differences in beta-diversity reflect both compositional shifts and increased inter-individual heterogeneity of the gut microbiome (Supplementary Figure S2).

The patients with obesity showed marked differences in composition compared to the control group. At the phylum level, the relative abundance of *Bacteroidota* was significantly lower in the obesity group, whereas *Firmmicutes* was no significant different between two groups (Figure 2D), which was in line with previous studies (18). At the genus level, the abundances of *Bacteroides*, *Phocaeicola*, *Roseburia* and *Parabacteroides* were reduced (Figure 2E). Conversely, patients with obesity exhibited increased abundances of the potential pathobionts including *Escherichia*, *Blautia*, *Mediterraneibacter* and *Klebsiella* (Figure 2E). These findings were in line with the abundance of phylum *Pseudomonadota (Proteobacteria)* increased in the obesity group (Figure 2D).

Differential species abundance analysis after adjusted sex and age revealed substantial microbial shifts between two groups (Figure 2F). Notably, among these taxonomy, *Streptococcus mutans* as known to produce ImP was markedly enriched in. We also found that *Lactobacillus gasseri*, *Streptococcus gallolyticus*, *Streptococcus infantarius*, *Streptococcus mutans*, *Streptococcus parauberis* and *Parvimonas micra*, which has the ability to produced imidazole propionate with “Y”- or “M”-UrdA homologs (19), were enriched in the obesity group. This finding was in agreement with our metabolomic data. However, ImP biosynthesis is classically attributed to *urdA*, comparison of total *urdA* gene numbers between groups revealed no significant difference (Supplementary Figure S3). Finally, correlation analyses between Imp, histidine concentrations and these differential species to explore potential microbioal and metabolic associations (Figure 2G). The results showed that *Lactobacillus gasseri* and *Streptococcus infantarius* were positively correlated with circulating ImP levels, along with *Limosilactobacillus fermentum*, *Fusobacterium* var*ium*, *Paeniclostridium bsordelli* and *Romboutsia hominis*, the latter of which was previously reported to be associated with ImP (20).

### 3.4 Multi-omics network linking gut microbiota, metabolome, and clinical parameters in obesity

**Figure 3:**
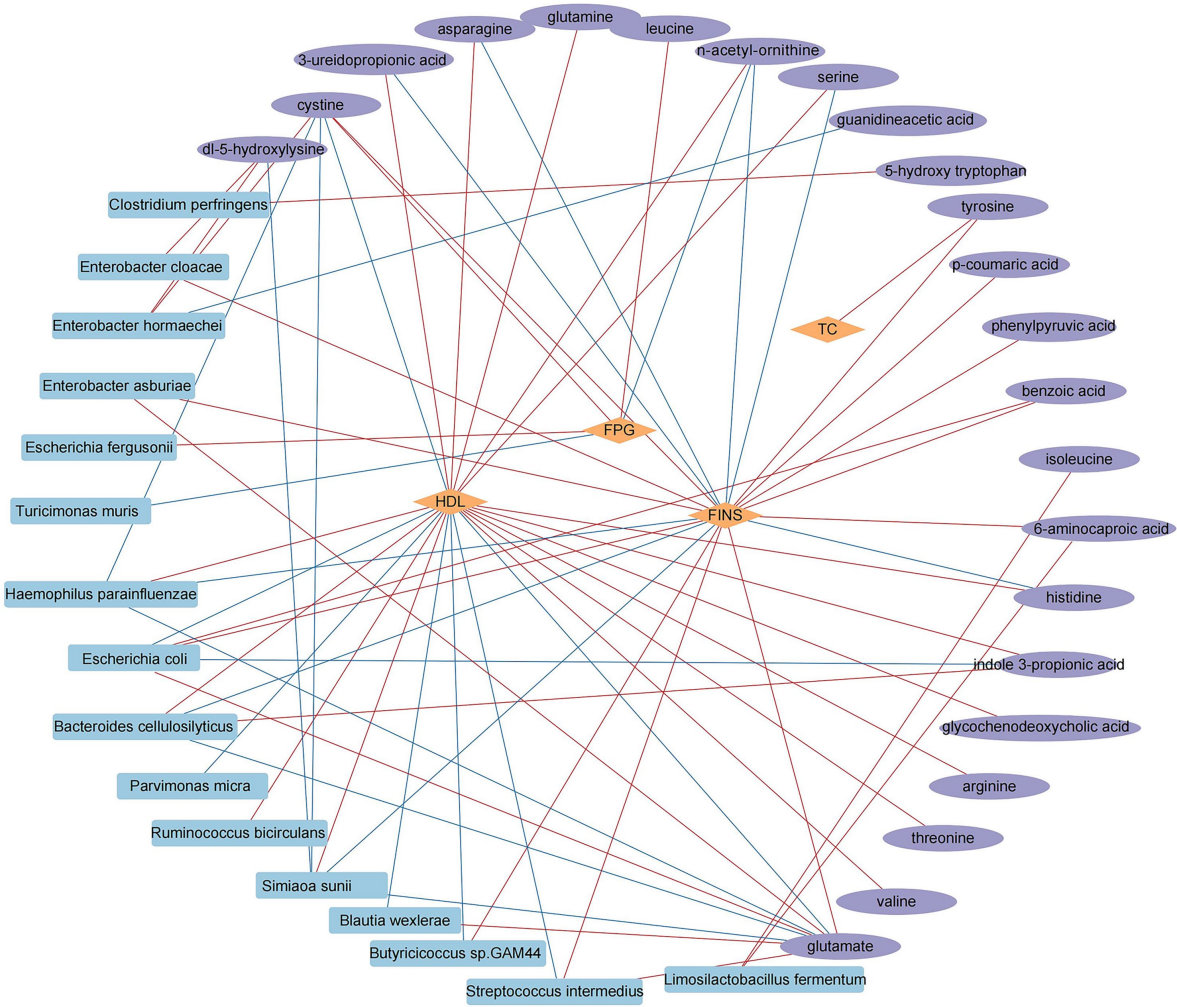
A muti-omics integrative correlation network. The network illustrates the interconnected relationships among significantly altered microbial species, metabolic features and clinical indices. Nodes are color-coded by category: blue for gut microbes, purple for metabolites, and orange for clinical metabolic parameters. Edges represent significant Spearman rank correlations (|*r*| ≥ 0.3, FDR < 0.05). Red edges indicate positive correlations, while blue edges indicate negative correlations. The width of the edges is scaled to the absolute value of the correlation coefficient (|*r*|), representing the strength of the association.

To characterize the interrelationship among gut microbiota, circulating metabolites, and clinical parameters in obesity, we conducted a muti-omics correlation network using Sperman correlation analysis. After applying stringent filtering criteria (|*r*| ≥ 0.3, FDR < 0.05), the network comprised 68 edges connecting 16 microbial species, 23 metabolites, and 4 clinical indicators (Figure 3). Based on node connectivity analysis, the clinical indicators HDL and FINS were the hub nodes with the highest connectivity in the entire network. At the metabolite level, glutamate and cystine were the main hubs in the metabolite layer. At the microbiota level, Simiaoa sunii and *Escherichia coli* had the highest connectivity.

Glutamate showed the strongest positive correlation with FPG (*r* = 0.702) and the strongest negative correlation with HDL (*r* = −0.741). At the microbial level, the depleted genera *Bacteroides cellulosilyticus* (*r* = −0.454), *Haemophilus parainfluenzae* (*r* = −0.431), and *Simiaoa sunii* (*r* = −0.441) were all negatively correlated with glutamate, while the enriched species *Escherichia coli* (*r* = 0.408), *Streptococcus intermedius* (*r* = 0.400), *Blautia wexlerae* (*r* = 0.313), and *Enterobacter asburiae* (*r* = 0.326) showed a positive correlation. Cystine (*r* = 0.390) and leucine (*r* = 0.301) were positively correlated with FPG, while n-*α*-acetyl-l-ornithine showed an inverse association (*r* = −0.300). Tyrosine was the sole metabolite positively correlated with TC (*r* = 0.310).

FINS reflects broad metabolic dysregulation. Beyond glutamate, FINS was positively correlated with cystine (*r* = 0.422), phenylpyruvic acid (*r* = 0.413), p-coumaric acid (*r* = 0.409), tyrosine (*r* = 0.406), benzoic acid (*r* = 0.332), 6-aminocaproic acid (*r* = 0.310). Conversely, 3-ureidopropionic acid (*r* = −0.401), asparagine (*r* = −0.397), histidine (*r* = −0.363), and serine (*r* = −0.357) were negatively correlated with FINS. At the microbial level, *Haemophilus parainfluenzae* (*r* = −0.446), *Bacteroides cellulosilyticus* (*r* = −0.313), Simiaoa sunii Haemophilus (*r* = −0.331) showed negative correlation with FINS, while *Butyricicoccus* sp. GAM44 (*r* = 0.343), *Enterobacter asburiae* (*r* = 0.311), *Enterobacter cloacae* (*r* = 0.325), *Escherichia coli* (*r* = 0.374), and *Streptococcus intermedius* (*r* = 0.341) was positively associated.

HDL reduction is associated with multiple amino acid depletions. HDL was positively correlated with asparagine (*r* = 0.505), 3-ureidopropionic acid (*r* = 0.503), serine (*r* = 0.395), indole-3-propionic acid (*r* = 0.366), threonine (*r* = 0.346), histidine (*r* = 0.342), and several other amino acids, all of which were concurrently reduced in individuals with obesity. Among microbial species, *Streptococcus intermedius* (*r* = −0.397), *Blautia wexlerae* (*r* = −0.383), *Escherichia coli* (*r* = −0.361), *Parvimonas micra* (*r* = −0.352), and *Butyricicoccus* sp. GAM44 (*r* = −0.320), which were accumulated in patients with obesity, showed negative correlation with HDL.

### 3.5 Dynamic alterations of imidazole propionate following bariatric surgery

**Figure 4:**
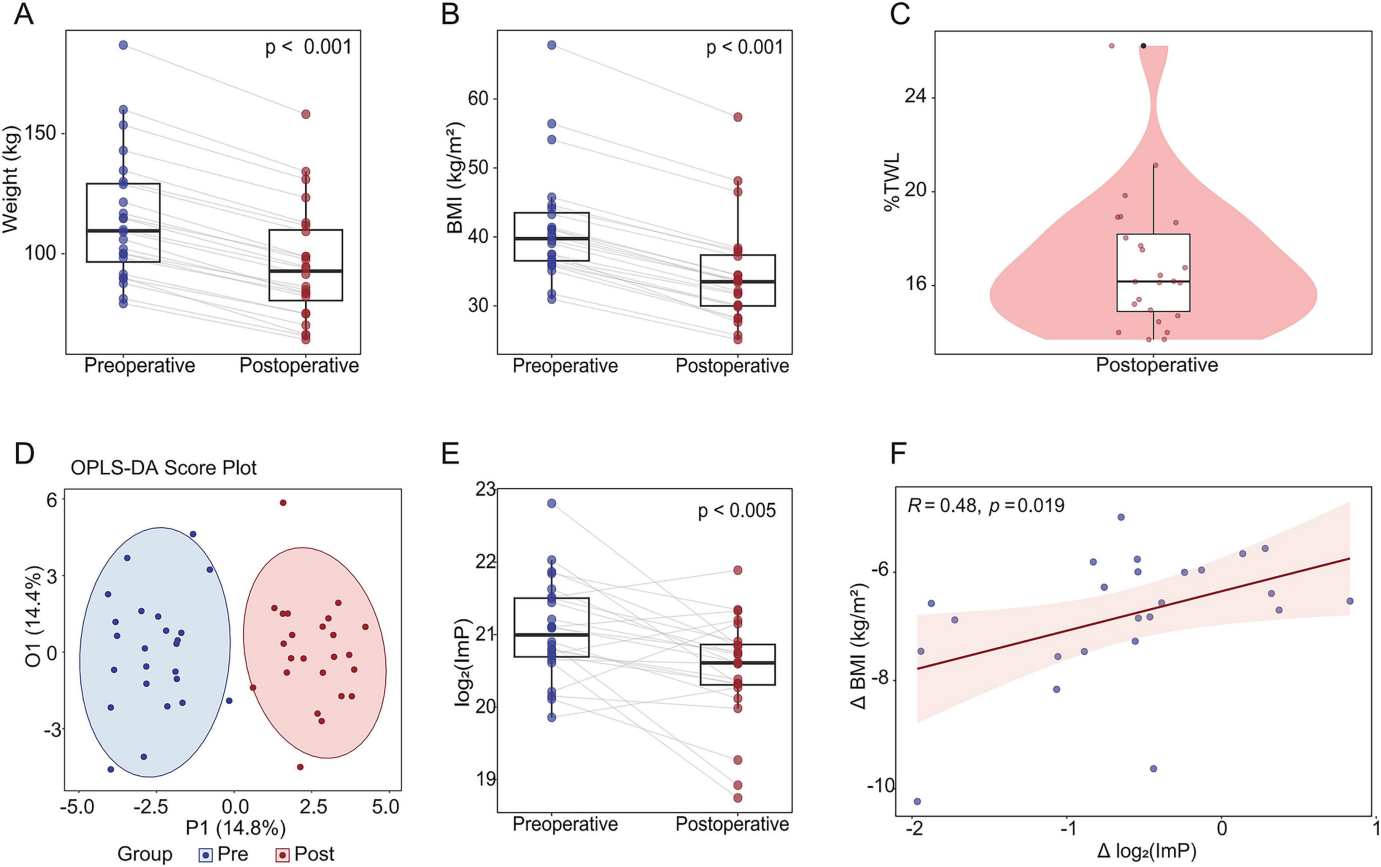
Bariatric surgery induces significant weight loss and a reduction in circulating ImP levels. **(A)** Changes in weight after surgery. **(B)** Changes in BMI after surgery. Gray lines connect individual data points for each participant preoperative and 3 months postoperative. **(C)** Distribution of total weight loss percentage (%TWL) at the 3-month follow-up. **(D)** OPLS-DA score plot based on metabolites. **(E)** Changes in the level of log_2_ImP after surgery. **(F)** Correlation between the changes in ImP and BMI. Correlation coefficients and significance were determined using Spearman correlation analysis (*R* = 0.48, *p* = 0.019). Statistical significance was determined using the paired Wilcoxon signed-rank test for longitudinal comparisons **(A,B,E)**.

Among the 91 subjects previously recruited, a subset of 24 patients underwent laparoscopic sleeve gastrectomy. Plasma samples and clinical parameters were collected at baseline and 3 months postoperatively. As expected, surgery induced significant weight loss and metabolic improvement. Paired statistical analysis showed a dramatic reduction in BMI and weight (*p* < 0.05) (Figures 4A,B). The median total weight loss percentage reached approximately 16.5% at the 3-month follow-up (Figure 4C). Beyond weight loss, significant improvements were observed in fasting plasma glucose, liver enzymes (Table 2).

**Table 2:** Comparison of clinical and metabolic characteristics before and after surgery.

Variable	Group		*p*-value^2^
	Preoperative *N* = 24^1^	Postoperative *N* = 24^1^	
Age	30.79 ± 6.53		
Sex			
Female	18 (75.0%)		
Male	6 (25.0%)		
Weight (kg)	114.58 ± 26.64	95.93 ± 23.94	0.014
BMI (kg/m^2^)	41.05 ± 8.26	34.60 ± 7.46	0.007
TC (mmol/L)	5.29 ± 1.12	5.55 ± 0.95	0.386
TG (mmol/L)	1.65 ± 0.67	1.26 ± 0.42	0.134
HDL-C (mmol/L)	1.11 ± 0.20	1.34 ± 0.72	0.743
LDL-C (mmol/L)	3.68 ± 1.03	3.58 ± 1.01	0.014
FPG (mmol/L)	5.87 ± 1.49	5.03 ± 0.53	0.001
ALT (U/L)	54.46 ± 34.15	25.88 ± 21.78	0.001
AST (U/L)	34.12 ± 18.67	21.38 ± 8.55	0.005
GGT (U/L)	46.38 ± 29.16	21.17 ± 18.62	<0.001

1 Mean ± SD or *n* (%), as appropriate.

2 *p*-value were derived from Paired *t*-test for continuous variables and Person’s chi-square test for categorical variables. A two-tailed *p*-value < 0.05 was considered statistically significant.

To characterize the systemic metabolic shift, we performed OPLS-DA analysis, which revealed a distinct separation between preoperative and postoperative metabolic profiles (Figure 4D). We found that only two metabolites serine and valine were increased after surgery, but many metabolites like tyrosine, lysine, alanine were decreased (Supplementary Figure S4). Most importantly, the plasma ImP levels were also significantly decreased after surgery (*p* < 0.05) (Figure 4E).

To determine if this reduction was mechanistically linked to clinical outcomes, we performed a correlation analysis on the changes of these parameters. We found that the magnitude of ImP reduction was positively correlated with the reduction in BMI (*R* = 0.48, *p* = 0.019) (Figure 4F). These results suggest that the restoration of the ImP reduction is closely associated with the weight loss and metabolic benefits achieved through bariatric surgery.

## 4 Discussion

Our study corroborates the established metabolic signature of obesity, marked by elevated circulating levels of BCAAs and aromatic amino acids (AAAs) (15, 21), while also unveiling a novel and microbiota-driven imbalance specific to histidine metabolism. We observed a divergent trend characterized by depleted levels of histidine concomitant with a significant elevation of its gut microbiota-derived catabolite, Imidazole propionate (ImP). Previous studies demonstrated that plasma ImP is elevated in individuals with T2D compared to normoglycemic controls; however, those studies employed BMI-matched designs to isolate the effect of glycemic status, leaving unanswered whether ImP is elevated in obesity per se (19, 22). Our study found that plasma ImP levels not only correlates positively with BMI but also increased progressively with obesity severity, with significant elevations observed in moderate, severe, and very severe obesity compared to healthy controls. Given that ImP has been previously linked to impaired glucose tolerance and cardiovascular risk (23, 24), its positive correlation with BMI in our study, independent of major confounding factors, reinforces its potential role as a mediator linking gut microbial activity to host metabolic dysfunction. Supporting this, we found that an enrichment of several species carrying “Y”- or “M”-UrdA homologs were positively correlated with circulating ImP.

The dose response relationship between ImP and obesity severity is particularly noteworthy, as it suggests that ImP may not only function as a biomarker but also actively drive obesity progression. The progression of obesity promotes gut dysbiosis that favors ImP-producing species, and the resulting elevation in ImP may in turn exacerbate metabolic dysfunction, thereby reinforcing further weight gain. The systemic elevation of ImP observed in our obesity cohort likely drives this vicious cycle through a multifaceted pathological landscape involving distinct molecular mechanisms. Firstly, ImP functions as a potent activator of the p38γ/p62/mTORC1 signaling pathway, which promotes the degradation of insulin receptor substrate (19), thereby directly contributing to the peripheral insulin resistance characteristic of obesity. Secondly, recent evidence identifies ImP as a critical immunomodulatory ligand that bind to the imidazoline-1 receptor (I1R) on myeloid cells (23), a pathway that may further exacerbate the chronic low-grade systemic inflammation characteristic of obesity (25). Beyond these two primary axes, ImP can cross the blood–brain barrier and accumulate in the hypothalamus, where it dampens neuronal reactivity and perturbs the excitatory/inhibitory balance (26). This neuro-modulation facilitates stress-induced emotional eating and maladaptive coping behaviors, potentially creating a vicious cycle of hyperphagia that is often observed in patients with severe obesity (27, 28). It is worth noting that while ImP generally promotes metabolic dysfunction, recent studies suggest it may inhibit the PPAR pathway to reduce lipid accumulation in mature adipocytes (29). This apparent discrepancy regarding lipid handling likely reflects tissue-specific pleiotropy (e.g., adipose tissue vs. immune system) or stage-dependent responses, underscoring the complexity of ImP as a systemic signaling molecule.

Metagenomic analysis confirmed significant dysbiosis in individuals with obesity, characterized by reduced species richness and a shift from beneficial commensals to potential pathobionts. The reduction in SCFA-producers, such as *Bacteroides* and *Roseburia*, likely contributes to compromised intestinal barrier function and systemic inflammation (30, 31). Our muti-omics correlation network illustrates how these compositional shifts translate into specific metabolic consequences. Glutamate as central metabolic node negatively correlated with HDL and positively correlated with FINS, which is consistent with previous studies reporting that elevated plasma glutamate is associated with insulin resistance, obesity, and nonalcoholic fatty liver disease (32). Notably, *Bacteroides cellulosilyticus*, a multifunctional carbohydrate-degrading bacterium capable of enhancing anti-inflammatory IL-10 production (33), was negatively correlated with both glutamate and FINS, suggesting that this species may exert a modulatory role in obesity-related metabolic dysregulation and potentially possess probiotic relevance. Indole-3-propionic acid (IPA), a tryptophan-derived microbial metabolite with well-documented anti-inflammatory and intestinal barrier-protective properties (34–36), was correlated positively not only with potential probiotic—*Bacteroides cellulosilyticus*, but also with HDL, further supporting a potential microbiota–metabolite axis linked to lipid metabolism. *Haemophilus parainfluenzae*, traditionally recognized as a pathogen associated with meningitis, pneumonia, and epiglottitis (37, 38), is also a commensal organism of the gastrointestinal tract (39). In the present study, it was the most strongly connected microbial node in relation to clinical indicators, though its precise metabolic relevance in the context of obesity remains to be elucidated.

Bariatric surgery remains the most effective intervention for severe obesity and T2DM (40–42), leading to rapid metabolic remodeling beyond mere weight loss, such as the reduction of BCAAs and AAAs and tryptophan and its downstream kynurenine metabolites (43). To our knowledge, this is the first study to demonstrate that LSG was associated with a significant and rapid decrease in plasma ImP within 3 months post-operation, and the magnitude of reduction correlated positively with BMI loss. Given the previously reported links between ImP and insulin signaling, inflammatory regulation, and vascular metabolic dysfunction (22–24, 29), the postoperative decline in ImP may represent one component of the broader metabolic remodeling induced by LSG. However, because the longitudinal surgical cohort was relatively small and the present study was observational, these findings should be interpreted as associative rather than causal.

Despite these insights, several limitations should be acknowledged. First, detailed dietary intake data were not available. Because diet strongly influences gut microbial composition (22) residual confounding by dietary habits cannot be excluded. Although all blood samples were collected after overnight fasting and participants with recent antibiotic, probiotic, health product, or nutritional supplement use were excluded, future studies incorporating food frequency questionnaires, 24-h dietary recalls, or controlled dietary records are needed to clarify the diet–microbiota–histidine metabolism–ImP axis in obesity. Second, the longitudinal surgical cohort was relatively small and limited to a three-month follow-up. Therefore, the postoperative decrease in ImP should be interpreted as an exploratory observation rather than evidence of causality or mediation. Larger longitudinal cohorts with longer follow-up and paired postoperative microbiome profiling are required to validate the dynamic changes in ImP after bariatric surgery. Finally, although several putative ImP-producing taxa were enriched in individuals with obesity and correlated with circulating ImP, total annotated UrdA-related gene abundance did not differ significantly between groups. This suggests that circulating ImP may be influenced not only by total UrdA gene abundance, but also by specific carrier taxa, substrate availability, microbial activity, and host metabolic clearance. Future functional studies are needed to causally validate the role of ImP-related microbial features in obesity.

## Data Availability

The data presented in this study can be found in the GSA (Genome Sequence Archive) repository (https://ngdc.cncb.ac.cn/gsa) under accession no. CRA075929.
