# Supplementary material for: Gut microbiota-derived imidazole propionate is associated with obesity
Source: Front Nutr. 2026 Aug 3;13:1861257. doi: 10.3389/fnut.2026.1861257 (PMC13479131; doi:10.3389/fnut.2026.1861257)
Supplement: Supplementary file 1 [file Supplementary_file_1.docx]

Supplementary Fig.1 Clinical correlation heatmap. Heatmap showing Spearman correlation coefficients between the differential metabolites (adjusted *p* < 0.05) and key clinical parameters, including BMI, total cholesterol (TC), triglycerides (TG), high-density lipoprotein (HDL), and fasting plasma glucose (FPG). P-values were corrected using the BH method. Asterisks denote statistical significance (**p* < 0.05, ***p* < 0.01).

Supplementary Fig.2 Comparison of total urdA gene abundance between the obese and control groups. The Wilcoxon rank-sum test indicates no statistically significant difference between the two groups.

Supplementary Fig.3 Volcano plot of serum metabolite changes before and after surgery. Each point represents a metabolite. The x‑axis shows the log₂ fold change (post‑/pre‑surgery), and the y‑axis shows the –log₁₀ of the *P*‑value from paired Wilcoxon signed‑rank tests. Metabolites with false discovery rate (FDR) < 0.05 are considered statistically significant after Benjamini‑Hochberg correction. Significant metabolites with log₂FC > 1.2 are colored red, those with log₂FC < 1.2 are blue; non‑significant metabolites are gray. Data were derived from paired pre‑ and post‑operative samples (n = 24 pairs).
